# Collaborative neurocardiology board meetings for decision-making in stroke care: a real-world experience

**DOI:** 10.1186/s42466-026-00464-w

**Published:** 2026-02-03

**Authors:** V. Mafael, T. Buck, H. Stengl, S. Hellwig, M.G. Klammer, M. Endres, M. Reinthaler, F. Barbieri, H.J. Audebert, D.M. Leistner, U. Landmesser, W. Doehner, C. Skurk, J.F. Scheitz

**Affiliations:** 1 Department of Neurology with Experimental Neurology, Charité-Universitätsmedizin Berlin, Berlin, Germany; 2 Center for Stroke Research Berlin (CSB), Charité-Universitätsmedizin Berlin, Berlin, Germany; 3 German Centre for Cardiovascular Research (DZHK), partner site Berlin, Berlin, Germany; 4 German Center for Neurodegenerative Diseases (DZNE), partner site Berlin, Berlin, Germany; 5 German Center for Mental Health (DZPG), partner site Berlin, Berlin, Germany; 6 Deutsches Herzzentrum der Charité, Department of Cardiology, Angiology and Intensive Care Medicine, Berlin, Germany; 7 Department of Cardiology, Angiology and Intensive Care Medicine, Charité – Universitätsmedizin Berlin, corporate member of Freie Universität Berlin and Humboldt-Universität zu Berlin, Berlin, Germany; 8 Institute of Active Polymers and Berlin-Brandenburg, Center for Regenerative Therapies, Helmholtz-Zentrum Hereon, Teltow, Germany; 9 Department of Cardiology, Universitäres Herz- und Gefäßzentrum Frankfurt, Universitätsmedizin Frankfurt, Frankfurt am Main, Germany; 10 German Centre for Cardiovascular Research (DZHK), partner site Rhein/Main, Frankfurt am Main, Germany; 11 Berlin Institute of Health at Charité (BIH), Center for Regenerative Therapies, Berlin, Germany

**Keywords:** Brain‒heart teams, Collaboration, Stroke, Patent foramen ovale, Left atrial appendage closure

## Abstract

**Background:**

Interdisciplinary collaboration between neurologists and cardiologists is needed to provide state-of-the art stroke care and individualized decisions about secondary prevention strategies. This highlights the need to establish working structures for joint decision-making between cardiologists and stroke neurologists. At present, studies describing the real-world decision-making as part of such brain-heart teams are scarce.

**Methods:**

We established a structured heart–brain team approach with monthly virtual neurocardiology board (NCB) meetings within a neurovascular network. We conducted a retrospective analysis of the implementation phase of NCB meetings between 2021 and 2024. We describe the structure of board meetings, patient characteristics and therapeutic and diagnostic recommendations.

**Results:**

During the study period, 46 board meetings were held, and 255 patients were discussed. The number of referred patients increased from 32 in 2021 to 89 in 2024. The majority of patients were evaluated for patent foramen ovale (PFO) closure (*n* = 189, 74.1%, median age 56 years, 59.8% female) and left atrial appendage occlusion (LAAO) (*n* = 32, 12.5%, median age 83 years, 50% female). Further questions centered around other individual secondary prevention strategies. Among patients referred to discuss PFO closure, closure was not recommended in 43.4% of patients (*n* = 82), recommended depending on additional diagnostic measures in 13.8% (*n* = 26), considered optional in 19.6% (*n* = 37) and clrearly recommended in 23.3% (*n* = 44). Patients for whom it was not recommended were older, had more cardiovascular risk factors and were less likely to have a large PFO shunt. The main reason for the recommendation against closure was that the PFO was not considered causal for the stroke (80.5%). Among LAAO patients, participation in a randomized controlled trial was recommended in 68.8%.

**Conclusions:**

We successfully established and steadily expanded regular NCB meetings to provide a platform for interdisciplinary exchange and personalized stroke treatment, in particular discussion of indications for interventional procedures against cardiac embolism. Our approach may serve as a blueprint for similar collaborative approaches. Future studies are needed to assess adherence to recommendations and patient outcomes as this study lacks follow-up data.

## Background

Stroke is a leading cause of mortality and permanent disability globally, with an annual incidence of 1.1 million in the EU [20]. Stroke care requires close collaboration between stroke physicians and cardiologists given the strong interplay between the nervous system and the cardiovascular system [15, 17]. This interaction includes three major fields of neurocardiology: (I) cardiac work-up to identify cardioembolic sources of embolism, (II) the prevention and management of cardiovascular complications, and (III) individual secondary prevention strategies such as interventional closure of a patent foramen ovale (PFO) or left atrial appendage closure (LAAO)(Doehner et al., [2, 6]. Current guidelines for stroke prevention acknowledge the medical need for interdisciplinary evaluation of stroke patients. For example, the American Heart Association/American Stroke Association [7] guidelines state that ‘recommendations for secondary stroke prevention in a patient with a PFO should be based on joint input from a neurologist with expertise in vascular neurology and a cardiologist with expertise in PFO closure’. Likewise, the Canadian Best Practice Stroke guidelines state that ‘patients with a recent ischemic stroke suspected to be related to a PFO should have an evaluation by healthcare professionals with stroke and cardiovascular expertise’ [3]. While concepts of brain‒heart teams have been proposed and interdisciplinary meetings are increasingly integrated into clinical routine [4, 10, 18], uncertainty remains how interdisciplinary neurocardiology decision-making can be organized in an efficient way.

**Fig. 1:**
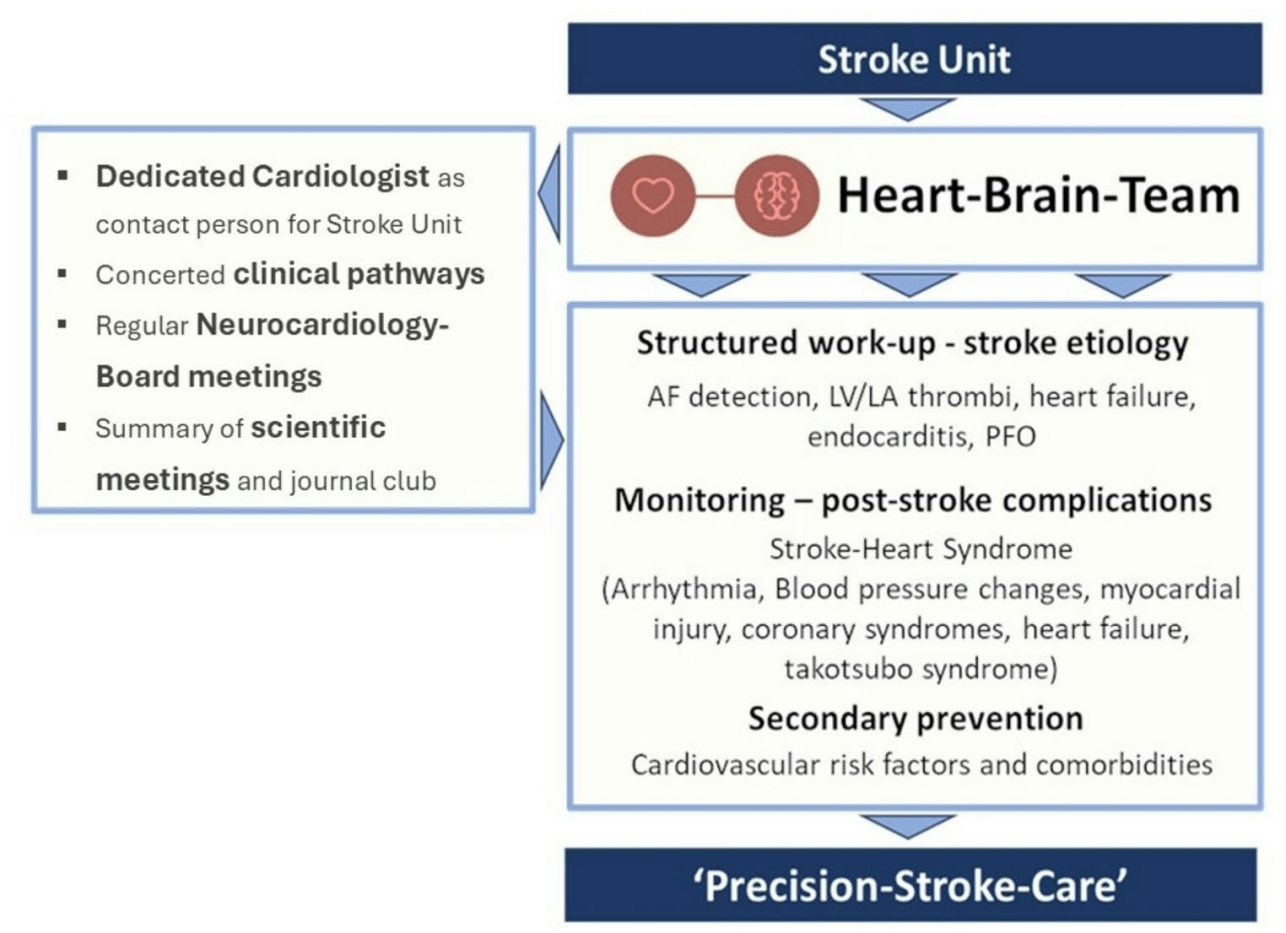
Brain-heart-team approach. Integration of dedicated cardiologists as a contact person for the stroke unit and concerted clinical pathways help facilitate a structured work-up of stroke etiology, optimize secondary prevention and treat poststroke complications. Part of this approach are regular neurocardiology-board meetings (NCB). Abbreviations: AF – Atrial Fibrillation; LV – Left ventricle; LA – Left Atrium; PFO – patent foramen ovale

In 2019, a patient-centered approach was implemented at the Charité Universitätsmedizin Berlin together with the ‘Deutsches Herzzentrum der Charité (DHZC)’, aiming to deliver precision stroke care by integrating clinical expertise from both specialties within a ‘Heart-brain team’ (Fig. 1). A key component of this approach is the regular scheduling of neurocardiology board (NCB) meetings—a monthly virtual platform discussing patients. Here, we report on the real-world experience during four consecutive years of NCB. We describe the structure and process of the implementation of the interdisciplinary NCB with a focus on board decisions concerning PFO closure in patients, referred to the board because of suspected PFO-associated stroke.

## Methods

### Description of the composition and process of the neurocardiology board

The Charité university hospital lies in the metropolitan area of Berlin (approximately 3.9 million inhabitants) and consist of three associated departments of neurology and includes the Deutsches Herzzentrum at the Charité (DHZC, with departments of cardiology at all campuses).

The NCB was originally established at Charité Campus Benjamin Franklin in 2019 to be conducted on a monthly basis. We intended to provide a platform for the structured interdisciplinary discussion of patients with uncertainties about optimal secondary prevention after stroke, particularly in patients considered for PFO closure or LAAO in patients with uncertainties about the risks and benefits of long-term anticoagulation treatment. Moreover, the NCB was intended to be a platform to discuss clinically relevant findings from recently published studies, present ongoing or planned collaborative projects, and revise or create interdisciplinary standard operating procedures.

While the first meetings were held in person, it was decided to conduct virtual meetings after a pause during the beginning of the COVID-19 pandemic. Since 2021, a regular monthly virtual NCB has been established. Initially, we referred to the board exclusively from the initiating hospital. Subsequently, referral to the board was extended to all three campuses of the Charité and centers within the associated neurovascular network comprising 5 external hospitals in Berlin and its suburban belt.

Patient referral to the board is conducted via secure email through a specific functional email address distributed to members of the neurovascular network and DHZC. The medical records of each submitted patient are comprehensively reviewed by a team of qualified stroke neurologists and supervised medical students using a prespecified set of variables considered relevant to address the respective question (including neuroimaging findings such as infarct patterns and cardiac imaging results, such as PFO risk features, vascular imaging data, laboratory data, and other relevant risk factors).

**Fig. 2:**
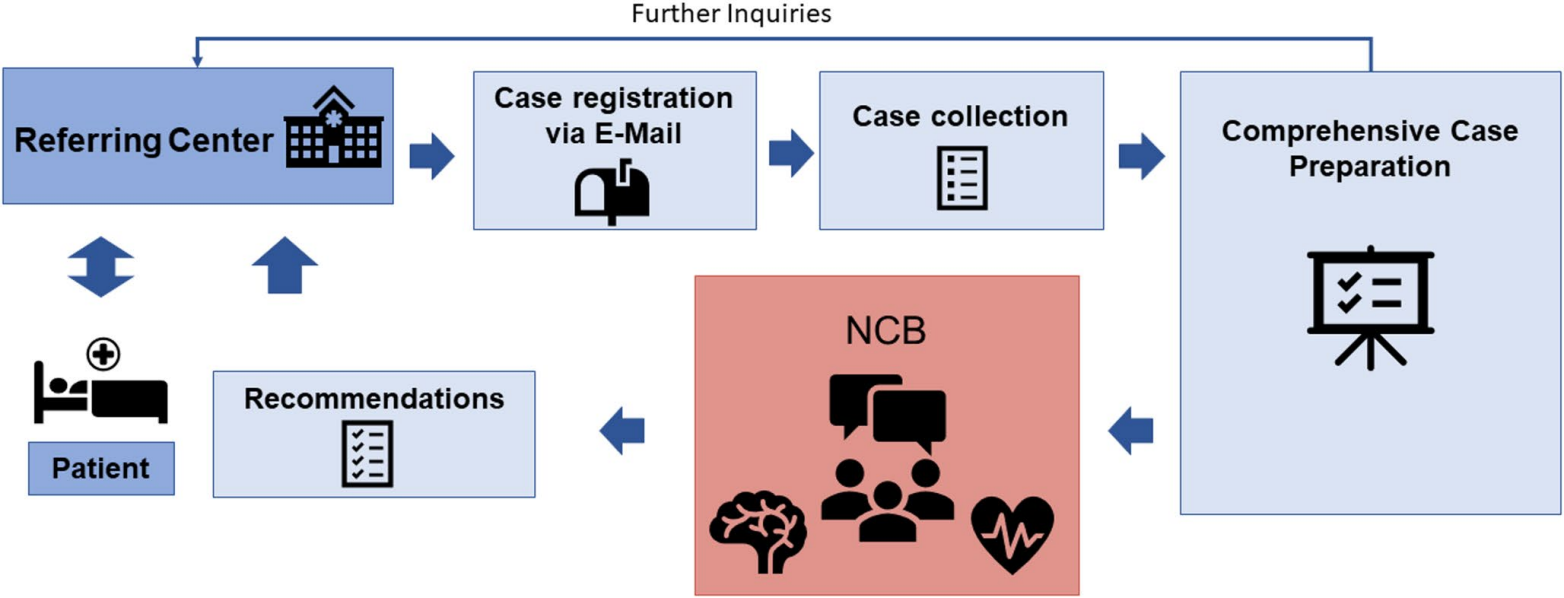
Schematic illustration of the organizational pathway of the neurocardiology board (NCB). Patients are referred via e-mail and, after comprehensive case preparation, are discussed in our online board meeting. Recommendations made by the board are communicated to the referring center, which informs the patients of the decisions and schedules further necessary steps (e.g., follow-up visits or interventions)

The participation of at least one board-certified neurologist with experience in the management of stroke and the participation of one board-certified cardiologist with expertise in interventional cardiology are mandatory. The virtual meetings are led by a designated moderator. At our site, this was performed by a stroke neurologist given the predominant referral of stroke patients to the NCB (JFS). Participation at the NCB is open not only to all involved physicians but also to physicians in training or medical students to provide teaching opportunities. Invitations to participate are sent via email approximately 14 days before the next scheduled meeting, containing the link for attendance as well as serving as a reminder for referring physicians. Cases are discussed separately, and individual recommendations are made upon consensus. A written protocol including the decision of the NCB is communicated to the respective referring physician via email or telephone. It is the responsibility of the physician, who refers a patient for discussion, to inform the respective patient about the board recommendations in an appropriate manner (Fig. 2).

### Criteria for patient referral to the NCB

The NCB was established as an open platform that allows referrals of all patients considered relevant or interesting for the neurocardiology community either because of unanswered clinical questions or teaching aspects. This included patients with uncertainties regarding the optimal secondary stroke prevention strategy, especially those considered for cardiac procedures like PFO closure and LAAO or patients with complex decisions concerning anticoagulatory treatment. In addition, we encouraged the referral of every patient considered interesting to the community, including those for teaching purposes and rare cases.

Owing to the uncertain benefits of PFO closure in certain populations, we formalized that stroke patients diagnosed with PFO and no other identified specific cause of the stroke should be referred to the NCB at low-threshhold, particularly those underrepresented in the seminal PFO closure trials (e.g. elderly patients or TIA patients or recurrent cryptogenic stroke). There were no formalized exclusion criteria for board submission.

### Collection of patient data

Patient data were collected from routine clinical records from all patients who were referred to the NCB between 2021 and 2024. A pre-specified set of variables was collected for each submitted patient depending on the reason for patient referral. Owing to the COVID-19 pandemic, after an inaugural meeting in 2019 and one meeting in January 2020, only seven meetings were held in 2020; therefore, these meetings were not included in the systematic collection of patient data. The scientific evaluation and analysis of the clinical data of the NCB work is covered by the Berlin legislation for hospitals (§ 25 Berliner Krankenhausgesetz) that approves the use of data derived during routine care and quality monitoring for scientific evaluations without requiring informed consent. The following data were recorded for the NCB reporting: the referring site/hospital, referring discipline, clinical facts of the case to be discussed (i.e. demographic data (age, sex), stroke-related characteristics (infarct pattern on cerebral imaging), and data concerning cardiovascular risk factors such as diabetes, hypertension, coronary heart disease, atrial fibrillation, hyperlipoproteinaemia and history of prior stroke or TIA) and clinical questions addressed to the NCB. Relevant information was gathered from the presentations from the board meetings that were created on the basis of available patient data, as well as the board protocols and patient medical records. For each patient referred for discussion of PFO closure, we also collected risk assessment scores such as the RoPE (risk of paradoxical embolism) score [5] and PFO-related risk factors (ASA, shunt size).

We further analyzed the performance of the NCB regarding number of cases discussed, duration of board meetings, number of participants and medical specialties and special topics during *n* = 13 exemplary board meetings chosen as a random sample between 05/2022 and 12/2023.

### Statistical analysis

Statistical analysis was performed via IBM SPSS Statistics (Armonk, NY, USA, Version 30.0.0.0). Data are shown as number (%) for categorical data or as the mean ± SDs or median and ranges and interquartile ranges (IQRs) for continuous data. The chi-square test was used to compare categorical data between groups. Student’s t test was performed to analyze differences in continuous data between groups. In addition, we calculated crude Odds Ratios (OR) and corresponding 95% confidence interval (95%-CI). A p value of < 0.05 was considered to indicate statistical significance. If data were missing, cases were excluded from group comparison of these variables. The study was conducted and reported in accordance with the STROBE guidelines (Strengthening the Reporting of Observational Studies in Epidemiology) [19].

## Results

### NCB characteristics and referral patterns

**Fig. 3:**
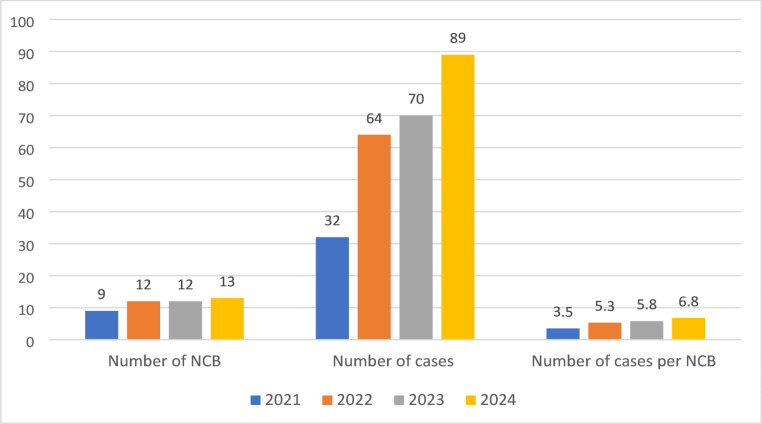
Development of patient numbers discussed at the neurocardiology board (NCB) between 2021 and 2024. The columns indicate the number of cases shown above the columns, whereas the years are indicated by color. A total of 255 patients were discussed during 46 NCB meetings between 2021 and 2024. A median of 5 patients (IQR 3–8, range 1–11) were discussed per meeting. The number of patients increased from 32 patients with 9 NCBs (3.5 ± 1,1 patients per board) to 89 patients with 13 boards in 2024 (6.9 ± 2,8 patients per board)

A total of 255 patients were discussed during 46 NCB meetings between 2021 and 2024. A median of 5 patients (IQR 3–8, range 1–11) were discussed per meeting. The number increased from 32 patients in 9 NCBs in 2021 (3.5 ± 1.1 patients per board) to 89 patients in 13 boards in 2024 (6.9 ± 2.8 patients per board, increase of 275%, Fig. 3).

Most patients were referred to the NCB from the coordinating site at the Charité Campus Benjamin Franklin (*n* = 186, 72.9%), 32 patients (12.5%) were referred from Charité Campus Mitte, and 27 (10.6%) patients were referred from Charité Campus Virchow. A total of 10 patients (3.9%) were referred from physicians or external (non-Charité) hospitals (e.g., from regional stroke centers that are part of the associated neurovascular network). The proportion of patients referred from other campuses or from external institutions increased from 0% in 2021 to 14% in 2022, 25% in 2023, and 47% in 2024.

Performance assessment of the NCB in an exemplary sample of 13 meetings conducted between May 2022 and December 2023 showed a mean duration of the NCB was 39 min (SD ± 15.5 min, range 22–56 min), constituting 7–8 min per discussed patient. On average, two board-certified cardiologists (median 2, IQR 1–2), with at least one specializing in interventional cardiology, and two board-certified neurologists, with at least one specializing in stroke medicine (median 2, IQR 1–3), were attending. Additionally, a median of three neurologists or cardiologists in training, as well as guests or medical students, were attending (IQR 2–5).

**Fig. 4:**
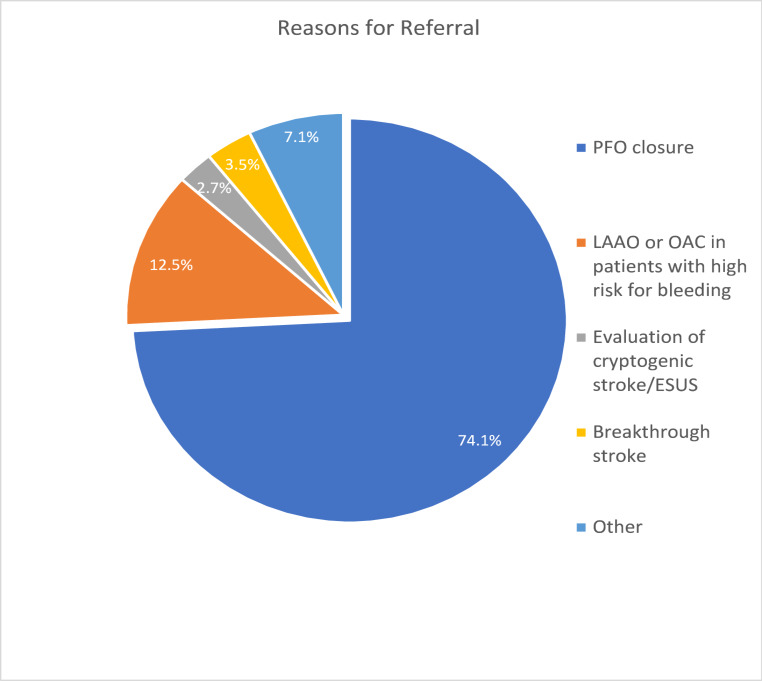
Reasons for patient referral to the neurocardiology board (NCB). In decreasing order, PFO closure (*n* = 189, 74.1%), LAAo or OAC treatment in patients with high bleeding risk (*n* = 32, 12.5%), breakthrough stroke (*n* = 9, 3.5%), evaluation of embolic stroke of undetermined source (*n* = 7, 2.7%) or other uncertainties about secondary stroke prevention (*n* = 18, 7.1%) were used. Abbreviations: PFO – patent foramen ovale, OAC – oral anticoagulant, LAAo – left atrial appendage occlusion, AF - atrial fibrillation, ESUS – Embolic Stroke of Unknown Source

The most common NCB referral reason was the treatment decision for suspected PFO-associated stroke, deciding on PFO closure vs. medical treatment (*n* = 189, 74.1%). In 32 patients (12.5%), the decision on LAAO vs. medical treatment was discussed in ischemic stroke patients with atrial fibrillation and high risk of bleeding (especially after intracranial hemorrhage (*n* = 18, 7.1%). Further topics were the need for advanced cardiovascular diagnostics, including prolonged cardiac monitoring in patients with embolic stroke of undetermined source (*n* = 7, 2.7%), optimal secondary prevention after stroke despite oral anticoagulation (OAC) in patients with known atrial fibrillation (‘breakthrough stroke’, *n* = 9 3.5%), secondary prevention in patients with intracardial thrombus (*n* = 3, 1.2%) and other discussions concerning individualized decisions in secondary stroke prevention and neurocardiology topics (*n* = 15, 5.9%. Fig. 4). The relative proportion of referrals for discussion of PFO closure decreased from 76.5% from 2021 to 2023 to 69.7% in 2024, whereas the topic of LAAO in patients with ICH or breakthrough stroke increased from 12.6% from 2021 to 2023 to 22.5% in 2024. Following an initial increase from 2021 (23/32) to 2022 (52/64), the overall annual number of patients referred to the NCB to discuss PFO closure remained stable over time, with the annual number of cases ranging from 52 to 62.

### Patient characteristics

The characteristics of the patients referred to the NCB are shown in Table 1. The most common qualifying events were ischemic stroke (*n* = 190, 74.5%), TIA (*n* = 28, 11.0%), and intracranial hemorrhage (*n* = 18, 7.1%). The median age of patients referred for PFO closure was 57 years (IQR 51–63, range 18–82), and 77 patients (40.7%) were female. The median age of patients referred to discuss LAAO versus medical treatment in case of high intracranial bleeding risk was 83 years (IQR 79–88, range 69–93) and 16 were female (50%). The median age of patients referred to discuss other topics was 69 years (IQR 53–76, range 32–95), and 12 patients (35.3%) were female.

**Table 1:** Characteristics of patients referred to the neurocardiology board

Characteristics	All patients (*n* = 255)	PFO closure (*n* = 189)			
		Recommended^1)^ (*n* = 107)	Not recommended (*n* = 82)	OR* (95%-CI)	*p* values
Sex, Female, n (%)	105 (41.2)	43 (40.2)	34 (41.5)	1.05 (0.58–1.89)	0.86
Age (yr.), Median (IQR)	60 (53–69)	56 (49–60)	61 (53–66)	0.95 (0.93–0.98)	< 0.01
Range (IQR)	18–95	18–74	22–82		
Age > 60 years, n (%)	128 (50.2)	29 (27.1)	44 (53.7)	0.32 (0.17–0.59)	< 0.01
**Qualifying Event**					
Stroke, n (%)	190 (74.5)	85 (79.4)	61 (79.3)	1.01 (0.49–2.05)	0.97
TIA, n (%)	28 (11.0)	14 (13.1)	10 (12.2)	1.08 (0.45–2.58)	0.85
Intracranial hemorrhage, n (%)	18 (7.1)	/	/		
Other, n (%)	19 (7.4)	6 (5.6)	7 (8.5)	0.86 (0.30–2.49)	0.79
**Risk factors**					
Coronary artery disease, n (%)	40 (15.7)	11 (10.3)	13 (15.9)	0.61 (0.26–1.43)	0.25
Hypertension, n (%)	153 (60.0)	42 (39,2)	56 (68.2)	0.3 (0.16–0.55)	< 0.01
Diabetes, n (%)	37 (14.5)	6 (5.6)	15 (18.3)	0.26 (0.09–0.71)	< 0.05
Hyperlipoproteinemia, n (%)	179 (70.2)	75 (70.1)	57 (69.5)	1.02 (0.55–1.92)	0.93
Known AF, n (%)	49 (19.2)	0 (0.0)	3 (3.7)	0.43 (0.35–0.50)	0.08
Prior Stroke or TIA^2)^, n (%)	76 (29.8)	22 (20.6)	23 (28.0)	0.66 (0.34–1.30	0.23
RoPE-Score ≥ 7, n (%)	/	42 (39.2)	12 (14.6)	3.76 (1.82–7.78)	< 0.01
Median RoPE score (IQR)	/	6 (5–7)	5 (4–6)	1.50 (1.24–1.81)	< 0.01
Large PFO^3)^, n (%)	/	94 (87.9)	63 (76.8)	2.32 (0.95–5.68)	0.06
ASA documented^4)^, n (%)	/	21 (19.6)	18 (21.9)	0.86 (0.41–1.78)	0.69

Patients referred to the neurocardiology board with the question of PFO closure are described according to final recommendations of PFO closure (i.e. PFO closure rejected versus closure considered at least optional, ‘can be considered’ or ‘should be done’). Abbreviations: TIA - transitory ischemic attack, PFO - patent foramen ovale, AF - atrial fibrillation, ASA – Atrial Septum Aneurysm, RoPE – Risk of paradoxic embolism, OR – Odds-Ratio, 95%-CI – 95% confidence interval. (1) PFO closure was at least recommended optionally (e.g., pending further prolonged cardiac monitoring), (2) in nine cases, there was no information available concerning prior stroke or TIA, (3) in nine cases, no information concerning PFO size was given (4) in 30 patients ASA characteristics were not available. * Higher ORs indicate a higher odds of recommendation of PFO closure

### NCB decisions in patients referred for potential PFO closure

A detailed analysis of NCB decisions was performed for patients referred for possible PFO closure (*n* = 189). PFO closure was not recommended for 82 patients (43.4%). The NCB clearly recommended PFO closure in 44 patients (23.3%, ‘should be done’). In 26 patients (13.8%), PFO closure was recommended depending on the results of additional diagnostic measures (e.g., prolonged cardiac monitoring to find atrial fibrillation prior to PFO closure), and PFO closure was considered optional (‘can be considered’, e.g., in the case of explicit patient preference) in 37 patients (19.6%).

Compared with patients with at least an optional recommendation for PFO closure, patients for whom PFO closure was not advised were more often older than 60 years (53.6% vs. 27.1%; OR = 0.32; 95%-CI: 0.17–0.59; *p* < 0,01) and had a greater median number of documented cardiovascular risk factors (2 versus 1; OR = 0.61; 95%-CI: 0.46–0.81; *p* < 0.01). Moreover, the RoPE score was less often ≥ 7 (14.6% vs. 39.2%; OR = 3.76; 95%-CI: 1.28–7.78; *p* < 0,01), and numerically the PFO shunt size was less often considered large (76.8% vs. 87.9%; OR = 2.23; 95%-CI: 0.95–5.68; *p* = 0,06). There was no difference in terms of the presence of ASA. In nine patients, the PFO size was not specified, and in 30 patients, the ASA characteristics were not available. Among all patients referred for discussion of PFO closure, we analyzed documented reasons for board decision against PFO closure (*n* = 82). In most patients, interdisciplinary case review by the NCB did not assume a causal role of the PFO (e.g., causes other than PFO considered more likely, *n* = 66, 80.5%), or the NCB did not confirm the suspected diagnosis of embolic stroke or TIA (*n* = 9, 11.0%). For 7 patients (8.5%), PFO closure was not recommended because of unfavorable patient characteristics, such as older age with poor functional status (e.g. modified Rankin Scale 5), limited life expectancy or a high risk for periinterventional complications.

Within patients aged ≥ 60 years, 73 were evaluated for PFO closure (including 35 aged ≥ 65 years, 47.9%). Among these patients, PFO closure was rejected in 44 (60.3%) patients. In 6 (8.2%) patients, PFO closure was recommended without further diagnostics and was considered optional in 23 (31.2%) patients. Additional prolonged cardiac monitoring prior to PFO closure was recommended for most of these patients (15/23, 65.2%). Out of 29 patients aged ≥ 60 years for whom PFO closure was recommended at least optionally, 26 (89%) had a large PFO, and in 8 (28%) patients, the presence of ASA was documented. Patients for whom PFO closure was rejected were older (median 65 years [IQR 63–71] versus 63 years [IQR 60–65]; OR = 0.81; 95%-CI: 0.69–0.94; *p* < 0.01) and had a greater median number of cardiovascular risk factors (3 vs. 1; OR = 0.51; 95%-CI: 0.28–0.93; *p* < 0,05).

### NCB decisions for patients referred for potential LAAO

The main diagnosis at referral for potential LAAO was intracranial hemorrhage in 18/32 patients (56.3%) and ischemic stroke or TIA in 14/32 patients (43.8%). Of these, reasons for referral were evidence of cerebral microbleeds (*n* = 9/14, 2 fulfilled Boston 2.0 criteria for cerebral amyloid angiopathy), hemorrhagic transformation (*n* = 4/14) or severe gastrointestinal bleeding (*n* = 1/14).

As a result of NCB discussions, a clear recommendation for LAAO was given (‘should be done’) in 2 patients (6.3%) and in 3 patients (9.4%) recommendation for LAAO was given based on patient preference (‘can be considered’). Participation in a randomized controlled trial was suggested in 22 patients (68.8%) including trials comparing LAAO versus best medical treatment (*n* = 19) or OAC versus no OAC (*n* = 3). LAAO was not recommended in 5 (15.6%) patients. In these patients re-starting OAC was recommended.

## Discussion

The need to establish collaborative team approaches between cardiology and neurology to foster interdisciplinary stroke care is increasingly recognized [1, 6, 16]. Here, we report data from a four year experience after implementing a monthly virtual NCB as part of a local ‘brain-heart team’ approach. Our findings demonstrate the feasibility and clinical value of conducting regular moderated case-based discussions among neurologists and cardiologists and reaching a consensus about individual treatment plans.

Despite their clinical relevance, few published models or frameworks exist that integrate the disciplines of neurology and cardiology. These have mostly been organized in the form of outpatient clinics where patients visit a cardiologist and neurologist at the same time [10] or first separately, followed by a joint consultation [18]. This approach has the advantage of directly involving patient preference during discussion. Another published concept is the use of in-person multidisciplinary team meetings [4, 11]. Our proposed NCB model uses monthly virtual meetings which bind fewer resources (i.e., staff, logistical effort, room requirements) offer a low threshold for attendance by involved physicians as part of their routine work and enable the attendance of residents in clinical training for teaching purposes. Because of such format, a NCB can be implemented relatively easily within a neurovascular network. The lack of patient attendance limits the understanding of patient preferences. The need for an additional visit with the referring physician to communicate the board’s recommendation may be another notable limitation.

We observed a constant increase in patient numbers between 2021 and 2024, with approximately 7 patients referred to the NCB per month and 80–90 patients per year, as of recently. Moreover, the numbers of patient cases submitted from physicians outside of the initiating hospital increased noticeably over time. Overall, this suggests the growing acceptance of our concept and underlines the increasing demand from referring physicians to provide such platforms. Our numbers are well in line with the capacity and reported demand of other published concepts at large comprehensive stroke centers [10, 18].

The majority of published similar multidisciplinary stroke-heart teams focused solely on the question of PFO closure [4, 11, 18], whereas others allowed for additional questions such as LAAO and discussion of cryptogenic ischemic strokes with a suspected cardioembolic etiology [10]. The discussion of one main topic has the advantage of a higher level of standardization. During our NCB meetings, PFO closure was the predominant reason for patient referrals, accounting for approximately 75% of patients. However, as of 2024, we increasingly discussed other topics, such as LAAO in patients with high bleeding risk and high risk for ischemic stroke [8, 12, 16] and the management of ischemic stroke despite OAC in patients with atrial fibrillation (“breakthrough strokes”) [13, 21]. This demonstrates that emerging topics can be adopted and integrated into an NCB and that NCBs can be more than a ‘PFO board’. Notably, the majority of patients referred to the board for the topic of LAAO were suggested for inclusion into an RCT, highlighting the highly specialized character of the board and its possible character as a tool to offer patients participation in ongoing RCTs.

We observed that PFO closure was not recommended in approximately 40% of patients, especially in patients aged 60 years or older. This is in line with existing reports of similar approaches to facilitate interdisciplinary discussion of patients with PFO-related stroke. Tariq and colleagues established a ‘Heart-Brain Clinic’ in Texas, USA, providing outpatient care for patients with PFO-related strokes. Compared with patients seen in the standard referral system, the authors observed fewer outpatient visits before the final decision was made (2 versus 3), and the decision was less likely to favor PFO closure (67.6% versus 94.9%) [18]. A retrospective analysis from Nijmegen in the Netherlands examined data from patients with PFO-related stroke or TIA between 2016 and 2021. A before-and-after comparison was conducted following the introduction of a ‘heart stroke team’ in June 2018 showing a higher rate of recommendations against PFO-closure after implementation (36% versus 0%) [4]. Data collected as part of a ‘Heart and Brain Team’ from the Tuscany area in Italy reported an even higher rate of recommendations against PFO closure over a 7-year period of 53% [10]. A study from Sweden described the results of a multidisciplinary PFO conference at a regional center in Gothenburg. Between 2006 and 2009, 311 patients were evaluated for PFO closure at this conference. Overall, the closure recommendation rate was approximately 45% and remained constant over the four years studied [11].

In our study, patients for whom PFO closure was not recommended, were older and had a more pronounced vascular risk profile, reflected by a lower RoPE score. The most common reasons for not recommending closure were the identification of an alternative or more likely cause of the stroke, or that the suspected diagnosis of a cerebrovascular event (stroke or TIA) was ruled out during the case discussion. These findings are in accordance with the observations of other similar approaches outlined above in which age, vascular risk profile, PFO Shunt size and RoPE-Score were identified as the main variables influencing board decision, and in which identification of an alternative cause of the stroke was described as the most common reason against decision for PFO closure. This highlights the importance of interdisciplinary consultation in the evaluation of suspected PFO-related stroke. We also noted that PFO closure seemed to be more likely to be recommended in patients with large shunt sizes. Nevertheless, documentation of shunt size and ASA was missing from echocardiography reports in some cases. Given that these PFO characteristics are key variables in deciding for or against interventional closure a standardized process of assessing echocardiography in case of suspected PFO associated stroke is desirable. Taken together, these observations suggest that the decision-making process in our NCB was balanced, standardized and mirrored current guideline recommendations [3, 7, 9, 14]. Moreover, the NCB offers a structure to agree on common reporting standards in medical reports that are required for decision-making.

Some limitations of our study need to be mentioned. An important limitation is the lack of follow-up data that would allow to analyze adherence to NCB recommendations and explore the effect on patient outcomes. It remains yet to be established whether the efforts to run an NCB translate into reduced rates of stroke recurrence and better long-term functional outcomes and whether such an approach is cost-effective. Multicenter prospective studies (e.g. designed as cluster-randomized trials) evaluating the impact of an NCB on occurrence of recurrent stroke, cardiovascular events and patient-centered outcomes over a well-defined follow-up could help strengthen the evidence for heart-brain-team approaches. Another limitation of our study is the absence of data comparing decision-making before and after NCB implementation. This restricts the ability to draw conclusions about differences in PFO closure recommendations as well as potential changes in decision-making processes over time. Since referral of cases was particularly encouraged in ‘uncertain’ cases, there is referral bias, leading to a preferential discussion of patients with high uncertainty in the NCB and may lead to a limited generalizability of our findings to the broader population routinely considered for PFO closure in clinical practice.

Finally, other benefits, such as the satisfaction of patients and participating physicians (improvement of ‘team-building’) and possible teaching aspects of NCBs, have not been investigated further.

## Conclusions

The successful implementation of an interdisciplinary NCB across different hospitals may serve as a potential blueprint for establishing interdisciplinary collaboration between neurologists and cardiologists in the patient-centered management of stroke patients, particularly those with suspected PFO-associated stroke or considered for LAAO. Our findings suggest that such collaborative efforts result in decision-making that is in accordance with guideline recommendations and allow enrolling patients in clinical trials. Future studies with follow-up data are required to assess whether adherence to NCB recommendations is achieved and whether long-term patient-related outcomes improve.

## Data Availability

The data are available from the corresponding author on reasonable request.

## Abbreviations

AF: Atrial fibrillation
ASA: Atrial septal aneurysm
ESC: European Society of Cardiology
IQR: Interquartile range
LAAO: Left atrial appendage occlusion
NCB: Neurocardiology Board
OAC: Oral anticoagulation
OR: Odds Ratio
PASCAL: PFO-Associated Stroke Casual Likelihood Score
PFO: Patent foramen ovale
RCT: Randomized Controlled Trial
RoPE: Risk of Paradoxical Embolism Score
SOP: Standard operating procedure
SVES: Supraventricular Extrasystole
TIA: Transient ischemic attack
95%-CI: 95% confidence interval
