# Correction: Collaborative neurocardiology board meetings for decision-making in stroke care: a real-world experience

**DOI:** 10.1186/s42466-026-00472-w

**Published:** 2026-03-17

**Authors:** V. Mafael, T. Buck, H. Stengl, S. Hellwig, M. G. Klammer, M. Endres, M. Reinthaler, F. Barbieri, H. J. Audebert, D. M. Leistner, U. Landmesser, W. Doehner, C. Skurk, J. F. Scheitz

**Affiliations:** 1 Department of Neurology with Experimental Neurology, Charité— Universitätsmedizin Berlin, Hindenburgdamm 30, Berlin, 12203 Germany; 2 Center for Stroke Research Berlin (CSB), Charité-Universitätsmedizin Berlin, Berlin, Germany; 3 German Centre for Cardiovascular Research (DZHK), partner site Berlin, Berlin, Germany; 4 German Center for Neurodegenerative Diseases (DZNE), partner site Berlin, Berlin, Germany; 5 German Center for Mental Health (DZPG), partner site Berlin, Berlin, Germany; 6 Department of Cardiology, Angiology and Intensive Care Medicine, Deutsches Herzzentrum der Charité, Berlin, Germany; 7 Department of Cardiology, Angiology and Intensive Care Medicine, Charité – Universitätsmedizin Berlin, corporate member of Freie Universität Berlin and Humboldt-Universität zu Berlin, Berlin, Germany; 8 Institute of Active Polymers and Berlin-Brandenburg, Center for Regenerative Therapies, Helmholtz-Zentrum Hereon, Teltow, Germany; 9 Department of Cardiology, Universitäres Herz- und Gefäßzentrum Frankfurt, Universitätsmedizin Frankfurt, Frankfurt am Main, Germany; 10 German Centre for Cardiovascular Research (DZHK), partner site Rhein/Main, Frankfurt am Main, Germany; 11 Center for Regenerative Therapies, Berlin Institute of Health at Charité (BIH), Berlin, Germany

**Correction: Neurological Research and Practice (2026) 8:4**

10.1186/s42466-026-00464-w

The name of the author W. Doehner was incorrectly given as 'W. Döhner' in the original version. Additionally, the affiliations for the authors M. Endres, M. Reinthaler, F. Barbieri, D.M. Leistner, U. Landmesser, W. Doehner and C. Skurk had to be corrected. The correct affiliations should read as follows: 

Mafael V1, Buck T1, Stengl H1,2, Hellwig S1,2, Klammer MG1,2, Endres M1,2,3,4,5, Reinthaler M6,7,8, Barbieri F6,7,8, Audebert HJ1,2, Leistner DM9,10, Landmesser U3,6,7, Doehner W2,6,11, Skurk C6,7, Scheitz JF1,2,3

1. Department of Neurology with Experimental Neurology, Charité—Universitätsmedizin Berlin, Berlin, Germany
2. Center for Stroke Research Berlin (CSB), Charité-Universitätsmedizin Berlin, Berlin, Germany
3. German Centre for Cardiovascular Research (DZHK), partner site Berlin, Berlin, Germany
4. German Center for Neurodegenerative Diseases (DZNE), partner site Berlin, Berlin, Germany
5. German Center for Mental Health (DZPG), partner site Berlin, Berlin, Germany
6. Deutsches Herzzentrum der Charité, Department of Cardiology, Angiology and Intensive Care Medicine, Berlin, Germany
7. Department of Cardiology, Angiology and Intensive Care Medicine, Charité – Universitätsmedizin Berlin, corporate member of Freie Universität Berlin and Humboldt-Universität zu Berlin, Berlin, Germany
8. Institute of Active Polymers and Berlin-Brandenburg, Center for Regenerative Therapies, Helmholtz-Zentrum Hereon, Teltow, Germany
9. Department of Cardiology, Universitäres Herz- und Gefäßzentrum Frankfurt, Universitätsmedizin Frankfurt, Frankfurt am Main, Germany
10. German Centre for Cardiovascular Research (DZHK), partner site Rhein/Main, Frankfurt am Main, Germany
11. Berlin Institute of Health at Charité (BIH), Center for Regenerative Therapies, Berlin, Germany

The original article has been corrected.

